# Flavonoid-Rich Extract of *Dissotis rotundifolia* Whole Plant Protects against Ethanol-Induced Gastric Mucosal Damage

**DOI:** 10.1155/2020/7656127

**Published:** 2020-03-31

**Authors:** Michael Buenor Adinortey, Charles Ansah, Benjamin Aboagye, Justice Kwabena Sarfo, Orleans Martey, Alexander Kwadwo Nyarko

**Affiliations:** ^1^Department of Biochemistry, School of Biological Sciences, University of Cape Coast, Cape Coast, Ghana; ^2^Department of Pharmacology, School of Pharmacy and Pharmaceutical Sciences, Kwame Nkrumah University of Science and Technology, Kumasi, Ghana; ^3^Department of Forensic Sciences, School of Biological Sciences, University of Cape Coast, Cape Coast, Ghana; ^4^Centre for Plant Medicine Research, Mampong, Akuapim, Ghana; ^5^Department of Pharmacology, School of Pharmacy, University of Ghana, Legon, Accra, Ghana

## Abstract

*Dissotis rotundifolia* is a plant in the family Melastomataceae. The methanolic extract of the whole plant is reported to be rich in C-glycosylflavones such as vitexin and orientin. Though there are several reports on the ethnomedicinal use of this plant extract in stomach ulcers, experimental-based data is unavailable. The drive for carrying out this research was to obtain data on the possible ameliorative effect of the whole plant extract of *Dissotis rotundifolia* (DRE) in gastric ulcerations induced by ethanol in Sprague Dawley (SD) rats. SD rats were pretreated with 100, 300, and 500 mg/kg of DRE for 14 days after which an ulcerogen-ethanol was administered. Gross examinations of the stomach lining and histological analysis of gastric lesions were carried out coupled with an assessment of the antioxidant activity of gastric mucosa using MDA, GSH, CAT, and SOD as indicators. The data suggested a significant attenuation in gastric mucosal damage in DRE-pretreated ethanol-induced gastric ulcer reflected in the antioxidant status. There was also a reduction or absence of hemorrhage, edema, and leucocytes infiltration in DRE-treated groups compared to the negative control group. DRE conserved glutathione (GSH) levels, reduced malondialdehyde (MDA) levels, and enhanced catalase (CAT) and superoxide dismutase (SOD) enzyme levels. The present study shows that DRE possess protective effects against ethanol-induced ulcer damage in the stomach of rats, which could be attributed to its antioxidant activity.

## 1. Background

Peptic ulcer disease (PUD) is a gastrointestinal tract disorder, affecting many people globally [1] with the most prevalent type being gastric ulcer. Apart from the main etiological agent which is *Helicobacter pylori*, certain factors have been recognized in its etiology in humans and this includes excessive alcohol consumption [2, 3]. Gastric ulcer is reportedly linked with alterations in physiological indicators such as reactive oxygen species (ROS) and nitric oxide (NO), *in vivo* antioxidant biomolecules and enzymes, and gastric acid oversecretion [3]. Alcohol abuse has been associated with gastric ulcers [4]. A systematic review published on this subject backs the assertion that excessive alcohol intake mediates the generation of reactive oxygen species, a known indicator of the disorder [5]. Though there are some antigastric ulcer medications such as the proton pump inhibitors (PIs) and histamine 2-receptor antagonists, the majority leave in their wake inimical effects such as bloating, diarrhea, shortness of breath, fatigue, nausea, dizziness, lactic acidosis, hepatotoxicity, kidney toxicity, and lactic acid intoxication thus limiting their usage [6]. Currently, the quest to unearth alternative and more efficient treatment therapies is imminent due to the fact that many natural bioactive compounds such as flavonoids and alkaloids had been isolated from medicinal plants and have been identified as potential antiulcer agents [7, 8].

**Figure 1:**
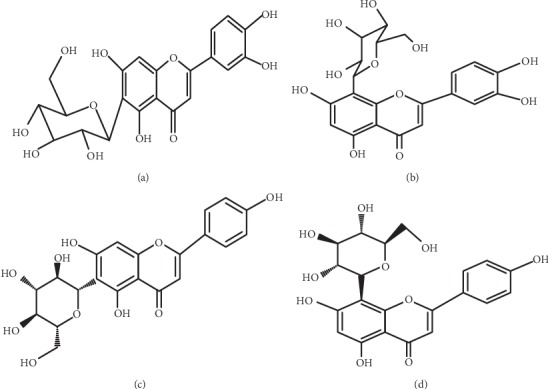
Structures of glycosylflavones compounds, (a) isoorientin (luteolin-6-C-*β*-glucopyranoside), (b) orientin (luteolin-8-C-*β*-glucopyranoside), (c) isovitexin (apigenin-6-C-*β*-glucopyranoside), and (d) vitexin (apigenin-8-C-*β*-glucopyranoside).

*Dissotis rotundifolia* (*D. rotundifolia*) is one such plant. It is a creeping plant commonly referred to as a pink lady in English. It is native to certain parts of Africa including Ghana. It has several ethnomedicinal uses including peptic ulcer. Evidence-based studies indicate that the whole plant is rich in C-glycosylflavones, namely, vitexin, isovitexin, orientin, and isoorientin [9], and structures are shown in Figure 1. Reports indicate that the extract of the plant possesses *in vitro* antimicrobial activity [10–12], *in vitro* antiradical effects, and H^+^/K^+^-ATPase inhibitory potential [13] and is nontoxic at 500 mg/kg bwt [14]. Although this plant is used in traditional medicine practices (TMPs) to manage peptic ulcer, there is scanty data to substantiate this ethnopharmacological relevance. This current study sought to evaluate the gastroprotective effect of a flavonoid-rich extract of *Dissotis rotundifolia* and identify possible antioxidant biomolecules interplaying in an ethanol-induced ulcer model.

## 2. Materials and Methods

### 2.1. Plant Collection and Preparation of Flavonoid-Rich Extract

The plant was gathered from the environs of the Kakum National Park in the Central region of Ghana. A Curator authenticated the plant with the voucher specimen number 107346 kept at the herbarium. The whole plant was carefully washed with clean tap water, shade-dried for 21 days, oven-dried at 40°C for 2-3 hours, and then ground into powder.

A two-step sequential method was employed in the extraction process as reported by Rath et al. [9] to obtain a flavonoid-rich extract. The first step involved putting a 1 : 10 W/V ratio of *Dissotis rotundifolia* powder and dichloromethane solvent in a flask. The flask was tightly corked and put on an IKA® KS260 basic shaker at a speed of 200 rpm for 2 days.

The mixture was filtered into a 2000 mL flask. After drying the residue at 40°C on a water bath, aqueous methanol (70%) was added, corked well, and put on a shaker at 200 rpm for another 2 days. The resultant mixture was later filtered. The filtrate was afterwards concentrated and dried at 40°C to get a brown coffee-colored extract of *Dissotis rotundifolia*. This was then labelled as *Dissotis rotundifolia* extract (DRE). The crude DRE of 8.3% yield was stored in a freezer at −20°C until ready for use.

### 2.2. Animals Used in This Experiment

Sprague Dawley (SD) rats of either sex (200–250 g) were used for this work. All experiments were conducted in accordance with “Principles of laboratory animal care” (NIH publication no. 86–23, revised 1985) in accordance with the National Institute of Health Guidelines for the Care and Use of Laboratory Animals [15]. Also, all protocols used in the study were approved by the Departmental Animal Ethics Committee. The animals used for the toxicity study were kept in stainless steel cages with softwood shavings as bedding material whereas the animals used for the ulcer experiment were kept individually in metabolic cages. All cages were kept under ambient temperature conditions (24 ± 2°C), relative humidity (60–70%), and 12 h light/dark cycle, and water and rat chow were available ad libitum. All animals used in this study were allowed to acclimatize to their new environment for at least two weeks with adequate water and food before the start of the experiment. However, preceding oral administration of DRE and standard drug-omeprazole, the animals were fasted for 24 hours overnight but were still allowed free access to clean water.

### 2.3. Acute Toxicity Assessment

The median lethal dose (LD_50_) of the extract was determined according to the method described by Ansah et al. [14]. DRE at the highest dose of 5000 mg/kg was used for the acute oral toxicity experiment. About sixty rats consisting of 30 male and 30 female rats each were placed into 6 groups of 10 rats each with an equal number of both sexes in each group. Group 1 that acted as normal control was administered distilled water only. Groups 2, 3, 4, 5, and 6 were given extracts at 10, 100, 500, 2500, and 5000 mg/kg bodyweight, respectively. Mortality was assessed within 24 h and observation continued for another 14 days. Also, changes in colors of the skin, eyes, fur, salivation, lacrimation, urinary incontinence, defecation, drowsiness, and tremors were examined. The stomach was isolated, cut opened, and observed for any gross pathological changes.

### 2.4. Gastroprotective Experiment

This experiment was done using ethanol (EtOH) as an ulcerogen. In traditional medicine practices (TMPs), the plant is used based on an oral administration of a decoction prepared from 40 g of the dry powdered herb in 1 L of water, taken at 5 mL 4X times daily [16] for a person of bodyweight 60–80 kg, and this translates approximately 300 mg/kg/day.

Subsequently, dosages of the extract used were chosen from results obtained from the acute toxicity study as well as translation of traditional dosage using the following equation:

(1) $$\begin{array}{c} \text{dosage}\left(\text{mg/kg}\right)=\frac{\text{volume of extract }\left(\text{mL}\right)\times \;\mathrm{conc}\text{ of extract }\left(\text{mg/mL}\right)}{\text{weight of animal }\left(\text{kg}\right)}. \end{array}$$

The extract was dispensed orally at 100, 300, and 500 mg/kg. The gastroprotective effect of DRE was studied in SD rats using the aforementioned dosages.

The experiment was carried out according to the method described by Adinortey et al. [17]. The SD rats were divided into six treatment groups with five animals in each group. Groups were pretreated for 14 days. Group I received 100 mg/kg of DRE; group II, 300 mg/kg of DRE; and group III, 500 mg/kg of DRE as pretreatment. Group IV received 30 mg/kg of omeprazole as pretreatment while groups V and VI served as negative and normal controls, respectively.

On the 13^th^ day after treatment with DRE and standard drug, animals in groups I–VI were fasted for 24 hrs prior to receiving the last oral dose of the extract and standard drugs. On the 14^th^ day, after 60 minutes of extract/drug treatment, all animals in groups I–VI were orally treated with 1 mL of absolute ethanol/200 mg/kg for the gastric ulcer induction. An hour later after ulcerogen administration, animals were sacrificed by cervical dislocation, and their stomachs were excised and analyzed.

#### 2.4.1. Macroscopic Examination of Stomach

The excised stomachs were washed thoroughly with saline solution to remove traces of gastric content and blood clots. They were spread on cardboard with the mucus surface upwards, avoiding corrugation, and were observed for ulcerations. Photographs were then taken.

#### 2.4.2. Gastric Ulcer Index

The total area of stomach and ulceration was traced by placing transparency on it. The stomach area and lesions/ulcers in the glandular part of the stomach were measured using a hand-held magnifying lens. The traced total area and the area of ulceration were each superimposed on a graph paper having a mm^2^ scale and measured. The total ulcerative area compared to the total area of each stomach was employed in the calculation of the relative area. The ulcer index of the relative area was determined from Table 1 reported by Ganguly [18].

(2) $$\begin{array}{c} \text{relative area}=\frac{\text{total mucosal area}}{\text{total ulcerated area}}, \end{array}$$

(3) $$\begin{array}{c} \text{percentage protective ratio}=\frac{\left(\left[\text{UI untreated}\right]\;-\left[\text{UI pretreated}\right]\right)\;}{\text{UI untreated}}\times \;100, \end{array}$$

where UI is the ulcer index

#### 2.4.3. Histopathological Evaluation

Stomachs of all rats in the experiment were excised and rinsed with saline solution to remove debris and blood clots. Sections of washed stomachs were cut and stored in 10% phosphate-buffered formalin. Excised tissues were washed in saline, fixed in Bouin's fluid, dehydrated in increasing concentrations of ethanol, and embedded in paraffin wax. Sections of tissues were then cut at 5 *μ*m with a rotary microtome, mounted on clean glass slides, and stained with haematoxylin. The tissues were then observed using an Olympus microscope and photographed by a chare-couple device camera at magnifications 100x.

#### 2.4.4. Determination of *In Vivo* Antioxidant Parameters

Stomach mucosal tissue scrapings obtained from rat stomachs were employed for the evaluation of antioxidant status specifically catalase (CAT), superoxide dismutase (SOD), reduced glutathione (GSH), and malondialdehyde (MDA).

The stomach mucosal scrapings were homogenized for 30 s in 0.9% cold saline (in 1 : 10 w/v) and centrifuged at 800 × g for 10 minutes and later at 12,000 × g for 15 minutes at 4^o^C. The supernatant collected containing the mitochondrial fraction was used for the estimation of antioxidant indices.

Reduced glutathione was determined according to the procedure by Ellman [19] and expressed as *μ*M/mg of protein. SOD activity was determined as described by Kakkar et al. [20], and the results were presented as mmol/min/mg protein. CAT was evaluated using the method described by Sinha, 1972 [21], and the results taken were expressed as units (U) of CAT mmol/min/mg protein. Lipid peroxidation (LPO) was estimated in gastric mucosal supernatant in terms of MDA content which was evaluated following the method reported by Ohkawa et al. [22], and the level of MDA measured was expressed as nmol MDA/g of tissue.

#### 2.4.5. Protein Determination

The protein content was measured according to the method reported by Bradford [23]. The parietal cell homogenate (1 mL) was measured into a test tube. 2 mL of the biuret reagent was added to the content in the test tube and gently swirled to mix. The content was then incubated at 37°C for 10 minutes, and the absorbance was read using a spectrophotometer at 540 nm. This was done in triplicate. A standard curve using bovine serum albumin (BSA) was prepared, and the protein concentration in the parietal homogenate was estimated from a standard curve.

### 2.5. Statistical Analysis

Data obtained was analyzed using GraphPad Prism, version 6.0 (GraphPad Software, San Diego, CA, USA). Descriptive and inferential statistics were used in the analysis of data. Values were presented as the mean ± standard error of the mean (SEM) in a table or graphical form for five animals. Groups were considered to be significantly different if *p* < 0.05. Bonferroni post hoc test was done where there was a significant difference for one-way ANOVA.

## 3. Results

### 3.1. Assessing Ulcer Indices in Rats

It was observed that pretreatment with DRE (100, 300, and 500 mg/kg) and omeprazole (30 mg/kg) significantly decreased ulcer indices in comparison with the negative control group (*p* < 0.05). No gross difference in ulcer indices was noticed among groups pretreated with 100 and 300 mg/kg of DRE and omeprazole (30 mg/kg) (*p* > 0.05). The percentage protection from ulcers was 70.23%, 95.96%, 54.55%, and 77.21% for groups pretreated with 100, 300, and 500 mg/kg of *Dissotis rotundifolia* and omeprazole, respectively (Table 2).

### 3.2. Gross Morphology of Stomach Epithelium after Inducing Ulcer in Rats

**Figure 2:**
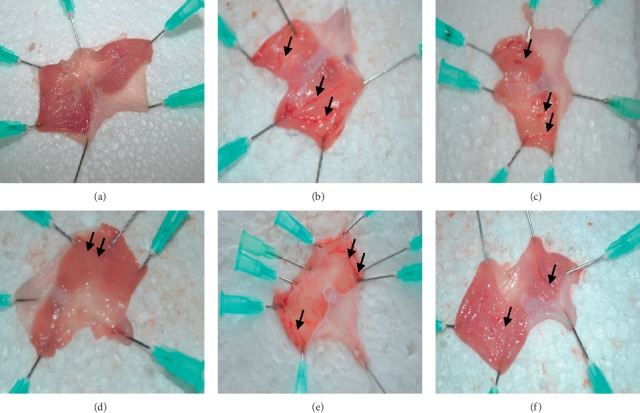
Representative appearance of the gastric mucosa of rats pretreated with DRE and omeprazole before ulcer induction with ethanol. (a) Rats pretreated with vehicle without ulcerogen (normal control). Normal gastric mucosa appearance. (b) Rats treated with only ulcerogen without any form of drug pretreatment. Severe injuries (black arrows) were observed in the gastric mucosa. Extensive visible hemorrhage necrosis was observed. (c) Rats pretreated with 100 mg/kg DRE followed by ulcerogen (ethanol). Moderate injuries (black arrows) to mucosa were seen. (d) Rats pretreated with 300 mg/kg DRE followed by ulcerogen (ethanol). Mild injuries to mucosa were observed. (e) Rats pretreated with 500 mg/kg DRE followed by ulcerogen (ethanol). Moderate injuries to mucosa were observed though not as 100 mg/kg DRE followed by ulcerogen (ethanol). (f) Rats pretreated with omeprazole followed by ulcerogen (ethanol). Injuries to mucosa are much milder as 300 mg/kg DRE.

Animals pretreated with omeprazole and different doses of DRE exhibited a significant reduction in gastric ulcer formation compared to negative controls (Figure 2). Severe hemorrhage was seen as red patches (black arrows) on gastric mucosae. Gastric ulcer seen as hemorrhage was markedly reduced in magnitude and severity in DRE and positive control pretreated animals (panel F). Meanwhile, there was severe ulcer formation in negative control animals (panel B).

### 3.3. Microscopic Assessment of Architecture of Stomach Epithelium after Inducing Ulcer

**Figure 3:**
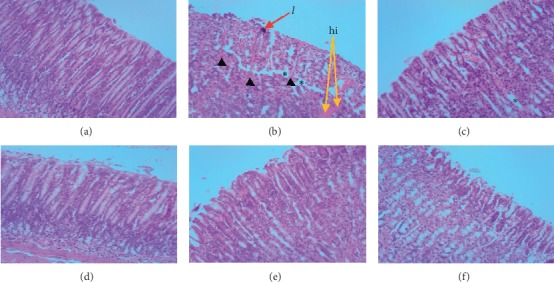
Photomicrographs represent histological sections of the gastric mucosa of rats pretreated with DRE and omeprazole before ulcer induction with ethanol. (a) Rats pretreated with vehicle without ulcerogen (normal control) showed normal gastric mucosa. (b) Rats treated with only ulcerogen (ethanol) without any form of drug treatment. The section displays extensive ulceration to the surface epithelium and with lesions penetrating deeply into mucosa accompanied by mark intra- and intermucosal hemorrhage (orange arrows; hi) with the presence of leukocyte (pink stains, see black arrowhead and red arrow) infiltration. Also, mucosal edemas (yellow asterisks) are seen within the mucosal layer. (c) Rats administered with 100 mg/kg DRE followed by ulcerogen (ethanol). The section shows reduced edema (yellow asterisk). (d) Rats administered with 300 mg/kg DRE followed by ulcerogen (ethanol). The section reveals normal gastric mucosa, which could be likened to the normal control group. (e) Rats administered with 500 mg/kg DRE followed by ulcerogen (ethanol) showed normal gastric mucosa. (f) Rats pretreated with omeprazole followed by ulcerogen (ethanol) showed normal gastric mucosa. H&E stain × 100.

Histological examination of mucosae in the ethanol-induced ulcer model revealed disturbance in the architecture of glandular and covering epithelium of the stomach (Figure 3). Histopathologic analysis of the groups pretreated with 300 mg/kg bwt of DRE (panel D) and 30 mg/kg bwt of omeprazole (panel F) showed mild damage to the gastric mucosa. The ethanol-induced ulcer caused severe disturbance of epithelium with mild edema and leukocyte infiltration (red arrow; l) into the submucosal layer (panel B).

### 3.4. Effects of DRE on Antioxidant Activity in Gastric Mucosa of Rats

The antioxidant characteristic of DRE was investigated *in vivo* using the ethanol-induced ulcer model. One-way ANOVA revealed a significant difference (*p* < 0.0001) in the levels of MDA, CAT, SOD, and GSH between control rats and those pretreated with DRE and OMP. Comparatively, rats pretreated with DRE only and OMP only exhibited no significant differences (*p* > 0.05) in the levels of all antioxidants measured. Therefore, an emphasis was placed on comparative differences between negative control (ethanol treated) and experimental (DRE and OMP pretreated) rats.

**Figure 4:**
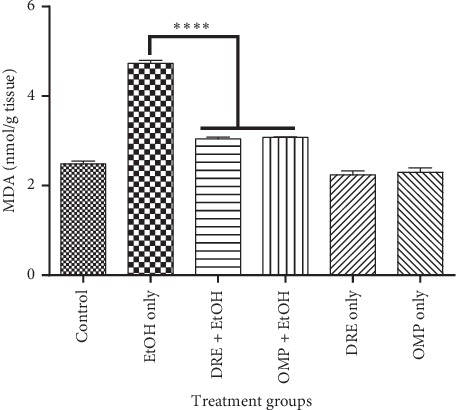
The effect of pretreatment groups on MDA levels. Pretreatment with DRE and standard drug (OMP) significantly reduced the MDA level compared with EtOH treated group. Data are shown as means ± SEM, ^*∗∗∗∗*^*p* < 0.0001.

In the present study, levels of MDA in ethanol-induced ulcer rats were high (Figure 4). Bonferroni post hoc test revealed that the level of MDA in gastric tissue of rats pretreated with DRE was significantly lower compared with that of negative control rats (*p* < 0.0001).

**Figure 5:**
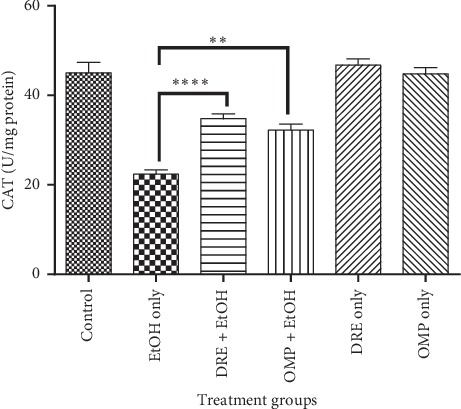
The effect of pretreatment groups on CAT levels. Pretreatment with DRE and standard drug (OMP) significantly increased the CAT level compared with EtOH treated group. Data are shown as means ± SEM, ^*∗∗∗∗*^*p* < 0.0001; ^*∗∗*^*p*=0.0016.

**Figure 6:**
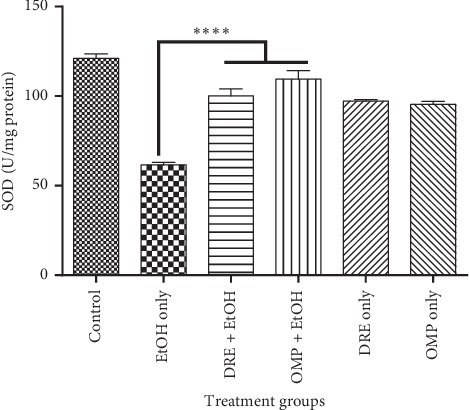
The effect of pretreatment groups on SOD levels. Pretreatment with DRE and standard drug (OMP) significantly increased the SOD level compared with EtOH treated group. Data are shown as means ± SEM, ^*∗∗∗∗*^*p* < 0.0001.

Again, an enhanced effect of DRE on CAT levels in the gastric mucosa was observed *in vivo* (Figure 5). Compared with negative control, there was a significant increase in CAT levels in DRE (*p* < 0.0001) and OMP (*p*=0.0016) pretreated rats. Similarly, DRE and omeprazole pretreated rats exhibited significantly higher SOD activities than the negative control group (*p* < 0.0001; Figure 6).

**Figure 7:**
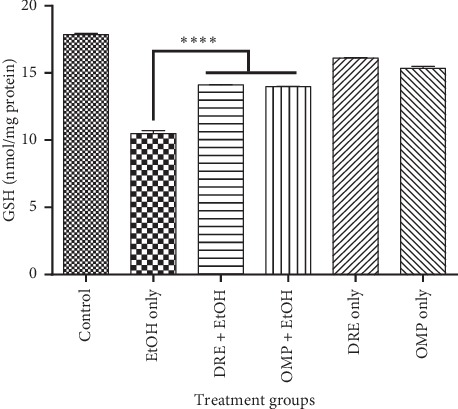
The effect of pretreatment groups on GSH levels. Pretreatment with DRE and standard drug (OMP) significantly increased the GSH level compared with EtOH treated group. Data are shown as means ± SEM, ^*∗∗∗∗*^*p* < 0.0001.

In addition, the influence of DRE pretreatment on GSH in gastric mucosal of rats was investigated. Ethanol treatment caused a significant reduction in GSH levels compared to all other groups (*p* < 0.0001). In contrast, DRE and OMP pretreatment boosted GSH levels to those similar to normal control rats (Figure 7). The results in this study indicate that DRE notably repressed the effects of ethanol on gastric GSH depletion.

## 4. Discussion

Ethanol is an etiological agent, which induces gastric mucosa lesions and petechial bleeding in the stomach [24]. It penetrates easily and rapidly into the gastric mucosa and causes membrane damage, exfoliation of cells, erosion, and sore formation. Ethanol-induced ulcer models are commonly used to study both the pathogenesis and possible therapies for human ulcerative diseases [17]. In this study, ethanol was used since it is one of the commonest etiological agents in peptic ulcers.

Medicinal plants play a vital role in the management of gastric ulcer and have been employed in the health care system in Ghana [16]. The ethnomedicinal use of *Dissotis rotundifolia* in Ghana for the management of gastric ulcer diseases with very little scientific evidence of efficacy necessitated this study. Acute toxicity studies on normal rats observed after 14 days did not show abnormal changes in the stomach lining even at a dosage of 5000 mg/kg, suggesting that DRE has no damaging effect on the integrity of the stomach.

According to a study by Glavin and Szabo [25], ethanol induces gastric ulcers by reducing the gastric mucosal blood flow and mucus production in the gastric lumen. Implicit disturbance in gastric mucus production as a result of alcohol exposure causes damage to the gastric mucosa, alters vascular permeability, and hampers free radical production. In this study, the negative control group treated orally with ethanol noticeably produced an expected characteristic zone of necrotizing mucosal lesions (Figure 2). Pretreatment with DRE drastically decreased ulcer index and also increased percentage protective ratios. These results indicate that DRE possesses an antiulcerogenic effect related to the cytoprotective activity.

Histological examination was performed to ascertain whether the induced ulcer affected mucosa layers and also assess the protective effects of DRE. Histopathological observation further confirmed the capacity of DRE to inhibit EtOH-induced gastric damage. Epithelium of rats pretreated with DRE showed a normal arrangement of gastric mucosal cells while ulcerogen-exposed rats demonstrated severe damage to stomach epithelium. Tissue sections of rats that were pretreated with 100 and 300 mg/kg of DRE and 30 mg/kg of omeprazole showed a marked reduction in the distortion of the mucosal layer and leukocytosis. This is indicative of a marked inhibition of gastric ulcers by DRE and omeprazole. The results from histopathological studies confirm that the plant extract possesses an antiulcer effect against ethanol in rats.

During ethanol metabolism, there is a release of superoxide anion and other free radicals which are usually cleared by free radical scavenging agents in order to avoid diseases such as peptic ulcers. Lately, many phytochemicals and their effect on suppressing free radicals' activity are being studied. A study by Adinortey et al. [13] shows that DRE possess inhibitory effects against free radicals, namely, OH, DPPH, SO, and NO *in vitro*. This necessitated this research to examine the effects of *Dissotis rotundifolia* extract in ethanol-induced ulcer model and also evaluate its effects *in vivo* on the antioxidant defense system. Scavenging free radicals are among the mechanisms reported to be involved in the healing of gastric ulcers [25]. Enzymatic defense system such as superoxide dismutase, glutathione peroxidase, catalase, and glutathione reductase and nonenzymatic defense antioxidant systems including reduced glutathione, *β*-tocopherol, vitamin C, and *β*-carotene play key roles against gastric tissue damage and toxicity by ROS [26].

Lipid peroxidation has been hypothesized to be one of the central features of ulcerogenesis [27]; hence, the effect of DRE on gastric lipid peroxidation was examined as an indicator of a defensive factor. Studies by Bradley et al. [28] in rats have shown that ethanol-induced ulcer is associated with increased LPO in gastric tissue. MDA, which represents an end product of peroxidation of polyunsaturated fatty acids and related esters within cell membranes, is considered as a reliable index of oxidative tissue damage [27]. In the present study, MDA levels of rats that were pretreated with DRE followed by ethanol exposure were found to be significantly lower than ethanol only exposed group (negative control). The MDA level in the ethanol only exposed group was seen to be higher than all other groups, denoting a rise in lipid peroxidation. The levels of MDA in mucosal tissue of rats pretreated with DRE were appreciably reduced when compared to the negative control group. This outcome indicates that DRE could recuperate the pathological condition of gastric ulcer disease by decreasing lipid peroxidation.

Superoxide dismutase enzyme is seen as the principal line of defense against the deleterious effects of oxygen radicals in the cell [26]. SOD converts the reactive superoxide radicals to H_2_O_2_, which, if not scavenged by catalase, can initiate an increase in LPO by generating hydroxyl radicals [26]. A reduction in SOD, CAT, and GSH levels can lead to the accumulation of these ROS and thus increased LPO and tissue damage. Meanwhile, an increase in SOD, CAT, and GSH levels can lead to decreased accumulation of these ROS and thus reduced LPO and subsequently less tissue damage in response to ethanol-induced oxidative stress.

In the present study, it was found that ethanol-induced ulcer rats showed decreased SOD and CAT levels in gastric mucosa. This could have led to increased lipid peroxidation and subsequently gastric tissue damage. Ethanol-induced suppression of SOD and CAT activities could have caused an increased flux of superoxide radical which may account for the increased lipid peroxidation in the negative control group in this study [29]. Meanwhile, there was an increase in these parameters (SOD, CAT) in DRE and omeprazole pretreated groups, which suggests a boost in the antioxidant system after pretreatment with DRE.

GSH is an antioxidant that plays pleiotropic roles, such as maintaining cells in a reduced state and functioning as an electron donor for some antioxidative enzymes such as glutathione peroxidase [30]. GSH level in patients with gastric ulcer is usually lower than that in the control group [31]. The depletion of glutathione levels in tissues leads to impairment of cellular defenses against ROS and consequently peroxidative injury. The significantly high levels of GSH observed in rats pretreated with DRE and omeprazole followed by ethanol compared to negative control ulcer group depict a boost in the antioxidant status as a result of drug administration. The observation in ethanol treated negative control group compared to DRE are consistent with other published reports [32]. The results in this study have not only reechoed the important relationship between SOD, CAT, and GSH levels and free radical induced oxidative stress in gastric tissues but have also provided evidence to support the ethnopharmacological relevance of *Dissotis rotundifolia* in managing gastric ulcer.

## 5. Conclusion

The data from the present study as demonstrated in ethanol-induced ulcer model show that flavonoid-rich extract of *Dissotis rotundifolia* whole plant displays gastroprotective property in rats. These findings corroborate the traditional use of the extract in managing stomach disorders. The results also indicate that *Dissotis rotundifolia* extract exhibits its gastroprotective effect in ethanol-induced mucosal injury by boosting antioxidant status through an increment in endogenous antioxidants, namely, GSH, CAT, and SOD and reducing the levels of MDA involved in lipid peroxidation.

## Figures and Tables

**Table 1:** The relative area and their corresponding ulcer index.

Relative area (mm^2^)	Ulcer index	Relative area (mm^2^)	Ulcer index
No ulcer	0	221–230	0.006
451–460	0.00001	211–220	0.007
441–450	0.00002	201–210	0.008
431–440	0.00003	191–200	0.009
421–430	0.00004	181–190	0.01
411–420	0.00005	171–180	0.02
401–410	0.00006	161–170	0.03
391–400	0.00007	151–160	0.04
381–390	0.00008	141–150	0.05
371–380	0.00009	131–140	0.06
361–370	0.0001	121–130	0.07
351–360	0.0002	111–120	0.08
341–350	0.0003	101–110	0.09
331–340	0.0004	91–100	0.1
321–330	0.0005	81–90	0.2
311–320	0.0006	71–80	0.3
301–310	0.0007	61–70	0.4
291–300	0.0008	51–60	0.5
281–290	0.0009	41–50	0.6
271–280	0.001	31–40	0.7
261–270	0.002	21–30	0.8
251–260	0.003	11–20	0.9
241–250	0.004	1–10	1.0
1–240	0.005	Perforation	–

Ganguly, 1969 [18]

**Table 2:** Effect of the whole plant extract of *Dissotis rotundifolia* (DRE) and omeprazole on ethanol (EtOH) induced gastric ulcers in SD rats.

Dosage (mg/kg bwt)	Ulcer index		% protection
Negative control		0.88 ± 0.020	—-
Normal control		—	100 ± 0.00
DRE	100	0.26 ± 0.080^ac^	70.23 ± 9.05
	300	0.04 ± 0.011^a^	95.96 ± 1.25
	500	0.40 ± 0.071^ab^	54.55 ± 8.03
Omeprazole	30	0.20 ± 0.090^ac^	77.21 ± 10.07

^a^Significant when a group was matched to the negative control group; ^b^significant when a group was compared to the omeprazole treated group; ^c^significant when a group was compared to 300 mg/kg bwt treated group.

## Data Availability

The data sets supporting the findings of this article are available in this write-up.
